# Fast robust dose calculation on GPU for high-precision ^1^H, ^4^He, ^12^C and ^16^O ion therapy: the FRoG platform

**DOI:** 10.1038/s41598-018-33194-4

**Published:** 2018-10-04

**Authors:** Stewart Mein, Kyungdon Choi, Benedikt Kopp, Thomas Tessonnier, Julia Bauer, Alfredo Ferrari, Thomas Haberer, Jürgen Debus, Amir Abdollahi, Andrea Mairani

**Affiliations:** 1 Division of Molecular and Translational Radiation Oncology, Heidelberg University Medical School, Heidelberg Institute of Radiation Oncology (HIRO), National Center for Radiation Research in Oncology (NCRO), Heidelberg, Germany; 2 Heidelberg Ion-Beam Therapy Center (HIT), Department of Radiation Oncology, Heidelberg University Hospital, Heidelberg, Germany; 3 Translational Radiation Oncology, German Cancer Consortium (DKTK) Core Center, German Cancer Research Center (DKFZ), Heidelberg, Germany; 4 BioDose and Personalized Radiation Oncology, National Center for Tumor Diseases (NCT), Heidelberg, Germany; 5 Heidelberg University, Faculty of Physics, Heidelberg, Germany; 6 National Centre of Oncological Hadrontherapy (CNAO), Medical Physics, Pavia, Italy; 7 University of Pavia, Department of Physics, Pavia, Italy; 8 Centre François Baclesse, Radiation Oncology, Medical Physics Department, Caen, France; 9 CERN, 1211, Geneva 23, Switzerland

## Abstract

Radiotherapy with protons and heavier ions landmarks a novel era in the field of high-precision cancer therapy. To identify patients most benefiting from this technologically demanding therapy, fast assessment of comparative treatment plans utilizing different ion species is urgently needed. Moreover, to overcome uncertainties of actual *in-vivo* physical dose distribution and biological effects elicited by different radiation qualities, development of a reliable high-throughput algorithm is required. To this end, we engineered a unique graphics processing unit (GPU) based software architecture allowing rapid and robust dose calculation. FRoG, Fast Recalculation on GPU, currently operates with four particle beams available at Heidelberg Ion Beam Therapy center, i.e., raster-scanning proton (^1^H), helium (^4^He), carbon (^12^C) and oxygen ions (^16^O). FRoG enables comparative analysis of different models for estimation of physical and biological effective dose in 3D within minutes and in excellent agreement with the gold standard *Monte Carlo (MC)* simulation. This is a crucial step towards development of next-generation patient specific radiotherapy.

## Introduction

Cancer centers equipped to treat radio-resistant and deep-seated tumors with particle beams are sprouting worldwide, promising more precise treatment delivery with superior tumor control and normal tissue sparing over conventional methods^1,2^. The enhanced biophysical anticancer properties associated with particle therapy exhibit a number of advantages such as overcoming hypoxia-related resistance and affording substantial critical organ dose sparing necessary for delicate cases seen in pediatric oncology^3,4^.

In contrast to photon irradiation that more than 50% of cancer patients receive during their course of disease, particle therapy with protons (^1^H) and heavier ions (such as ^12^C) is more sensitive to treatment uncertainties e.g. patient positioning, organ motion, range and beam delivery^5,6^. As the complexity of radiotherapy treatment techniques continues to rise, the need for fast and sophisticated treatment planning tools becomes more evident for both clinical and research purposes.

During facility startup and routine clinical operation, many particle therapy clinics will rely on commercially developed software throughout the treatment chain. To ensure the safety of patients and consistent quality of care, these softwares often arrive from the vendor in a precompiled format, which can delay testing and optimization of current and prospective physical and biological models for the clinic. Recent works undertake this issue by developing secondary systems in-house like matRad^7^ and FoCa^8^, educational MATLAB®-based tools for hadrontherapy treatment planning. Such platforms are usually benchmarked against their model center’s clinical treatment planning system (TPS), demonstrating good agreement. Other platforms for particle therapy include TRiP^9^ and Astroid^10^; however, the accuracy of such pencil-beam (PB) algorithms, the crucial element of every particle therapy dose calculation, should be under scrutiny in cases of severe patient geometry heterogeneity^11–13^. A more rigorous approach to development, by assessment and validation against the gold standard *Monte Carlo (MC)* simulation, may provide more insight into the predictive power of such analytical algorithms^14,15^.

When it comes to accuracy of dose calculation, attention to heterogeneous anatomy and its effect on beam evolution is a critical feature of a TPS. In conjunction with Gaussian parameterization, various computational methods to account for anatomical variability exist including point of interest (Syngo, Siemens Medical Solutions, Erlangen, Germany), dynamic splitting^16^, adaptive splitting^17^, kernel superposition^18^ and beamlet superposition^19^, each exhibiting a unique balance of calculation time and accuracy in heterogeneous conditions. Consequently, methods with longer calculation times typically offer more assured dose estimations in the presence of complex anatomy. As compact, high-performance hardware becomes more accessible, using graphics processing units (GPU) in place of the central processing units (CPU) can significantly reduce dose calculation runtimes^20–22^.

A GPU-based analytical dose calculation engine, FRoG, for the four ion beams (^1^H, ^4^He, ^12^C, and ^16^O) available at the Heidelberg Ion-beam Therapy Center (HIT) has been developed in-house, capable of accurate 3D dose computation within minutes. FRoG features a pencil beam model devised from *MC* simulation which explicitly accounts for interactions within the HIT beam applications and monitoring system (BAMS). The newly introduced GPU-based recalculation platform for particle therapy demonstrates excellent agreement with *MC*-calculated dose distributions in both homogenous scenarios and complex patient cases with strong anatomical heterogeneities.

**Figure 1:**
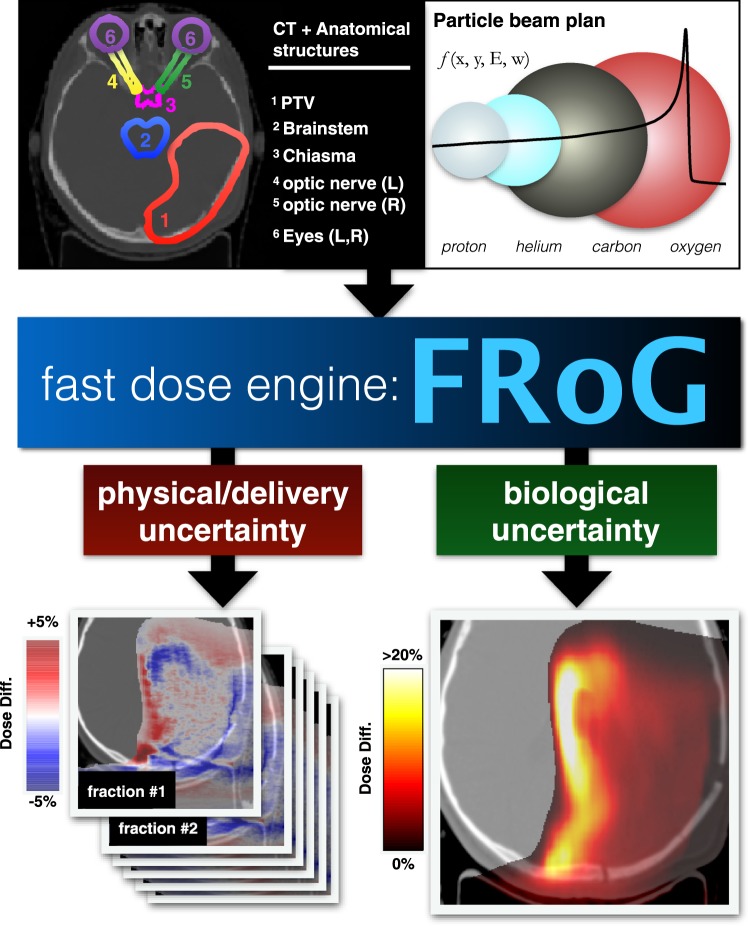
FRoG, Fast dose Recalculation on GPU, is a multipurpose platform for physical and biological dose calculation, functional for all four ions (^1^H, ^4^He, ^12^C, and ^16^O) available at HIT. By feeding in the necessary inputs, including patient specific (CT scan and delineated anatomical structures) and physical beam parameters (radiation quality, raster-scan spot coordinates, energy and fluence), FRoG can produce dose predictions which consider physical inter- and intra-fractional uncertainties (bottom left panel). It’s inherently open architecture makes possible the incorporation of biophysical models used clinically (e.g. constant RBE = 1.1 for protons), as well as those in research and development (variable RBE). A comparison between clinically implemented and data-driven models reveals distal biological dose variations of up to ~20% (bottom right panel).

Here, the dose calculation engine is validated, making way for clinic workflow integration and future retrospective study with the HIT patient database as shown in Fig. 1, such as linking physical, delivery or biological uncertainties in particle therapy to clinical outcome. Recent works provided evidence of variable relative biological effective (RBE) in a subset of patient follow-up MR scans, revealing a hidden complexity in biological track damage when using therapeutic proton beams^23^. With this in mind, a major goal of a fast computation engine like FRoG is to perform high-throughput patient calculations and use clinical outcome as an endpoint to develop data-driven biological effect models. Further efforts in FRoG development will focus on clinical accessibility and enhancement of computational performance, as well as implementation of research and clinical RBE models.

## Results

### Benchmark testing in homogenous geometry

**Figure 2:**
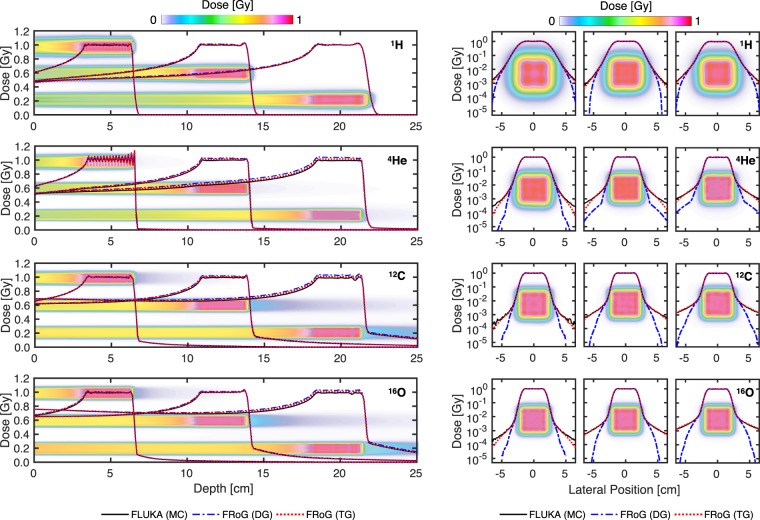
3 cm × 3 cm × 3 cm SOBP calculations in water for FRoG versus FLUKA at 50 mm, 125 mm and 200 mm depths for ^1^H, ^4^He, ^12^C, and ^16^O. Depth dose profiles (left) and lateral dose evolution in a logarithm scale (right) are presented. Background dose maps display cross-sections mid-SOBP for the three fields, scaled to the position (horizontal) axis. FRoG recalculations using both DG and TG parameterization are plotted, demonstrating improved agreement with *MC* when using higher order parameterization, especially for the heavier ions where the complexity of lateral dose evolution increases.

To gauge the performance of FRoG’s computational engine, physical dose was calculated for a set of spread-out Bragg peak (SOBP) plans in FRoG and compared with FLUKA *MC* simulation. Predicted (FLUKA) versus analytically calculated (FRoG) depth and lateral dose distributions for the four ions are shown in Fig. 2 for three 3 cm × 3 cm × 3 cm SOBPs centered at shallow (50 mm), mid-range (125 mm) and deep-seated (200 mm) depths. Dosimetric parameters for SOBP depth dose and lateral fall-off characterization are shown in Supplementary Table 1 for each ion and SOBP. Relative dosimetric parameters are defined as follows: range where dose falls to 80% of Bragg peak maximum (R_80_), tail-to-peak ratio (TPR), entrance-to-plateau ratio (EPR), dose fall-off from 80% to 20% of the Bragg peak (DFO_80/20_), lateral dose fall-off from 80% to 20% of the Bragg peak (LFO_80/20_), and dose homogeneity (H_D_), defined as the ratio between the lowest and the highest measured dose within the inner 80% of the SOBP. Absolute dosimetric parameters included average Dose ($\bar{D}$) and absolute percent difference ($| \% \triangle |$).

For all four radiation qualities, the absolute dose difference between FRoG and FLUKA was ≤2% for triple Gaussian (TG) parameterization and ≤4% using double Gaussian (DG) parameterization to model lateral dose evolution. Absolute percent differences in the target averaging over the three depths were 0.88(±0.21)%, 1.66(±0.28)%, 1.26(±0.71)% and 0.97(±0.57)% for TG, and 1.36(±0.06)%, 2.93(±0.92)%, 2.48(±1.42)% and 2.09(±1.25)% for DG, for protons, helium, carbon and oxygen ions, respectively. Dosimetric parameters such as R_80_, DFO_80/20_, LFO_80/20_ and H_D_ were similar for FRoG’s DG and TG calculation. Both were in good agreement with results from FLUKA *MC* simulation. However, EPR and TPR exhibited improved agreement with FLUKA calculation for higher order parameterization. These dosimetric improvements for TG manifest in better $\bar{{\rm{D}}}$ agreement with FLUKA, and hence a reduced $| \% \triangle |$. Protons yielded excellent agreement at all depths for TG and relatively good agreement for DG, with absolute dose difference well below <1% and <2% for TG and DG, respectively. Slight improvements in lateral fall-off agreement from DG to TG are visualized in Fig. 2. For the heavier ions, especially with carbon and oxygen, FLUKA and FRoG agreement improvements in $\bar{{\rm{D}}}$ and lateral profile agreement occur for the higher order Gaussian parameterization method, yielding a $\bar{{\rm{D}}}$ decrease in the target from ~4% to ≤2% for helium, carbon and oxygen ions. The dose maps in Fig. 2 visualize the physical benefits and tradeoffs of each ion i.e. improved target conformity with increasing mass, countered by a fragmentation tail.

### Validation in heterogeneous anatomy

**Figure 3:**
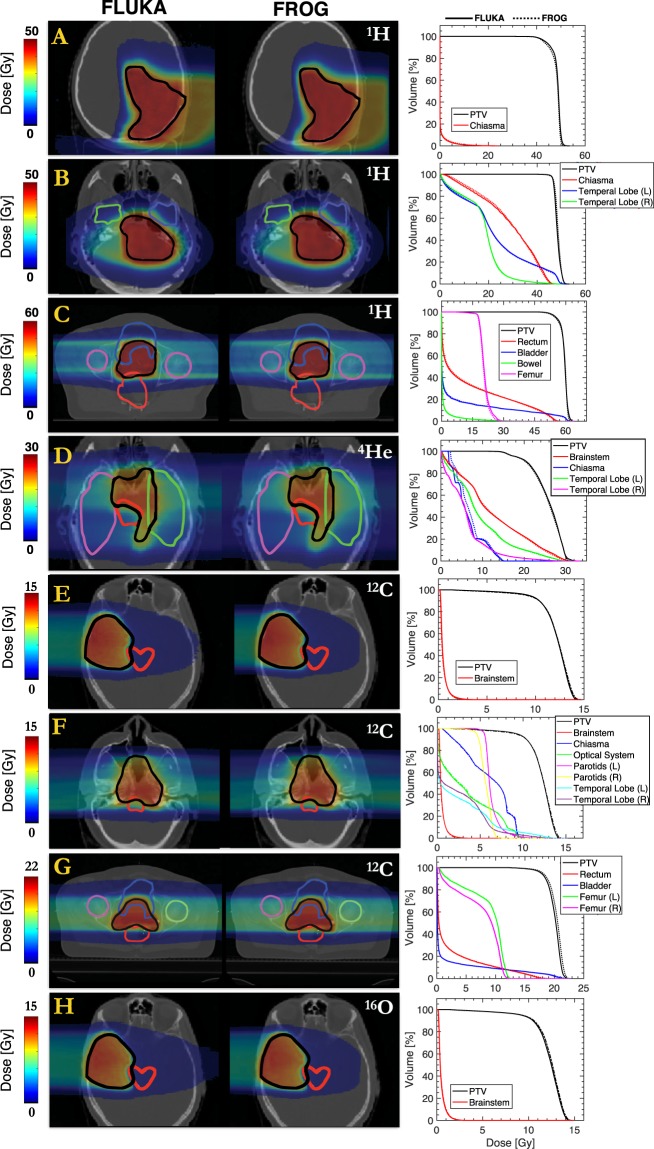
Physical dose recalculations using FLUKA and FRoG for clinical ^1^H (**A**–**C**) and ^12^C patient cases (**E**–**G**), as well as clinical-like treatments for ^4^He (**D**) and ^16^O (**H**). Corresponding DVHs for the PTV and OARs with clinically relevant doses are presented.

**Figure 4:**
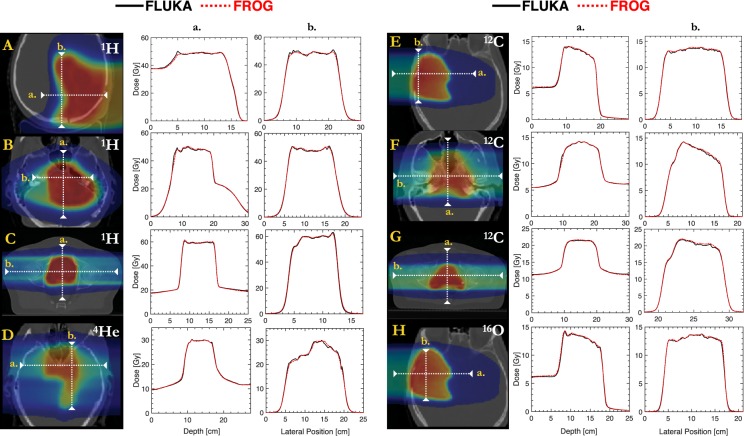
Line profiles for recalculated ^1^H (**A**–**C**), ^4^He (**D**), ^2^C (**E**–**G**) and ^16^O (**H**) ion beam plans.

Validation of dose calculation in patient cases followed a similar procedure of that described in AAPM TG-53^24^ and in the recent validation of the Monte Carlo Treatment Planning (MCTP) platform through line profile examination and dose volume histogram (DVH) assessment^25^. 3D dose distributions were compared to FLUKA *MC* simulation, previously validated against experimental results^26–28^. Benchmarking FRoG’s physics engine involved physical dose comparison with *MC* for all four ions. DVH statistics for FLUKA and FRoG are presented in Supplementary Table 2, detailing various dosimetric endpoints e.g. D_X%_, defined as the dose delivered to X% of a structure’s volume, such as D_95%_, D_50%_ and D_5%_ for the planning target volume (PTV) and organs at risk (OARs) reaching clinically relevant doses. Corresponding global percent difference (%Δ) for each statistic is displayed, normalized by the FLUKA predicted D_50%_ of the PTV (D_50%,PTV_) for each case. Corresponding dose maps and DVH plots are displayed in Fig. 3 for the four ions. Line profiles are displayed in Fig. 4.

## Discussion

The aim of project FRoG was to establish a user-friendly forward calculation method for both clinical and research purposes, providing *MC*-like accuracy with clinical TPS-like calculation speed. Table 1 presents a summary of the patient case calculations, including calculation times for FRoG and time gain factors in place of FLUKA *MC* simulation. Despite the use of ~300 CPUs in each *MC* calculation, large time gain factors are observed using an analytical code on a single GPU. Ideally, determining time gain factors of performing dose calculation on CPU versus GPU would require development of a CPU-based FRoG engine. Nevertheless, these gain time factors express the power of parallelized computing on the GPU.

**Table 1:** Patient Case Information.

HIT Patient Case Information										
ID	Particle Beam	Type	Fractions	Prescription	PTV Volume	CT Voxel Dimension	Dose Scoring Dimensions	Scan Spots	Calculation Time	Time Gain Factor
			#	[GyRBE]	[cc]	[mm^3^]	[mm^3^]	#	t	X
A	^1^H	Skull base Chordoma	30	1.8	220	0.6 × 0.6 × 3	2 × 2 × 2	8686	2m59s	87
B	^1^H	Chondrosarcoma	30	1.8	109	0.6 × 0.6 × 3	2 × 2 × 2	6759	2m16s	77
C	^1^H	Prostate	20	3.3	205	0.98 × 0.98 × 3	3 × 3 × 3	10670	3m02s	202
D	^4^He	Meningioma	29	1.8	54	0.6 × 0.6 × 3	2 × 2 × 2	3080	1m25s	103
E	^12^C	Chondrosarcoma	15	3.0	152	0.6 × 0.6 × 3	2 × 2 × 2	24471	3m07s	72
F	^12^C	Skull base Chordoma	15	3.0	219	0.6 × 0.6 × 3	2 × 2 × 2	27111	3m46s	115
G	^12^C	Prostate	20	3.3	217	0.98 × 0.98 × 3	3 × 3 × 3	20492	3m29s	237
H	^16^O	Chondrosarcoma	15	3.0	152	0.6 × 0.6 × 3	2 × 2 × 2	24471	2m43s	102

From left to right, columns list the patient case ID, particle species, disease diagnosis, number of fractions, fractional prescription [GyRBE], PTV volume [cc], CT grid size [mm^3^], dose scoring grid size [mm^3^], number of planned beam spots, FRoG calculation time and time gain factor (FLUKA to FRoG calculation time ratio). The FRoG times provided for each case were performed on a Tesla V100 NVIDIA card and an Intel Core i7 I7-7700K, 4.2 GHz, 16 GB RAM, while the FLUKA *MC* times involved a ~300 CPU cluster of Intel Xeon CPU E5-2683 16 core @ 2.1 GHz.

To gauge FRoG’s speed with commercially available platforms, forward calculations were performed with the three proton patient cases (A, B and C) using Syngo’s CPU-based PB algorithm (HIT’s current clinical system) and the RayStation (RaySearch Laboratories AB, Stockholm, Sweden) GPU-accelerated PB algorithm. Forward calculations were additionally repeated with FRoG employing RayStation’s approach to the PB algorithm by reducing PB splitting multiplicity to 19. Calculation times for patients A, B and C, respectively, for the three dose engines were as follows: 13.9 s, 7.8 s and 13.1 s for RayStation (NVIDIA Quadro P5000 card), 306 s, 240 s, and 295 s for Syngo (Intel Xeon CPU E5-2683 16 core @ 2.1 GHz), and 7.7 s, 7.8 s and 8.0 s for FRoG (NVIDIA Tesla V100 card). Since the three workstations use different hardware and due to Syngo’s use of an alternative lateral heterogeneity handling approach (point of interest) unlike FRoG and RayStation, direct comparison of calculation time is not feasible; however, considering typical CPU-based engine runtimes (~minutes)^7^, as demonstrated with Syngo, both GPU-based engines exhibit enhanced speed, which can be attributed to their parallelized dose kernel calculation procedures. Although such runtimes are clinically acceptable, the commercial analytical dose engines sacrifice accuracy for speed by inadequately describing the PB model in complex patient cases. FRoG counters with higher order lateral dose parameterization (TG) and a larger PB splitting multiplicity setting than clinical systems (see methods section).

When the clinic routinely encounters such dosimetric challenges, either due to heterogeneous anatomy or OARs close to the target volume, *MC* is often requested for verification; however, calculation times exceed the clinical standard, making *MC* unsuitable for the daily activity. Nonetheless, a validation of FRoG against the gold standard *MC* is the ideal test for accuracy.

In the homogenous cases (SOBP in water), FRoG’s forward calculation engine produces dose distributions in good agreement with *MC* for all four ions using TG parameterization ($|\triangle |$ ≤ 2%). Consequently, this level of agreement of FRoG and FLUKA is similar to results found in the recent validation of the FLUKA-based MCTP against physical measurements^25^. Calculation using TG’s higher order lateral beam profile model exhibited superior agreement with FLUKA simulation. Presented in Supplementary Table 1, maximum deviations occurred for the heavier ions, and deviations from FLUKA increased as a function of penetration depth. Improved agreement with FLUKA was realized when using TG over DG for all four ions. Regarding calculation using DG parameterization, proton SOBP cases saw the best dose agreement with FLUKA, with deviations <2% for all depths, while the heavier ions (helium, carbon and oxygen) saw larger but clinically acceptable deviations on the order of ~2–4%, increasing with target depth.

As anticipated, agreement degrades in more rigorous tests (greater depth) of the small target, signifying the limitations of Gaussian parameterization, even when a more sophisticated, time-intensive procedure is implemented. Despite DG and TG calculations exhibiting identical results for the lateral fall-off dosimetric parameters listed in Supplementary Table 1, Fig. 2 illustrates the advantage of using higher order parameterization for the heavier ions and its effect on the absolute dose difference in the entrance and plateau of the SOBP in Fig. 2, yielding ~2% reduction in $|\triangle |$ for the heavier ions. Overall, one finds that as particle mass and beam energy increase, the accuracy of the analytical calculation to describe the lateral dose penumbra decreases in comparison to *MC* methods. These results advocate application of higher order Gaussian parameterization for lateral beam evolution modeling in the next generation of analytical TPSs, starting with the heavier ions^11^. As presented in previous works^29^, similar methods of implementing TG for lateral beam profile modeling are already in clinical practice but, in contrast to FRoG’s engine, compensate for variable sigma of the second and third Gaussian by adjusting the weights as a function of depth while maintaining invariant widths^30,31^. Other recent works support the extension of non-Gaussian models to handle the complexity of lateral dose evolution in particle beams^32^.

In the heterogeneous setting of patient cases, FRoG exhibits good agreement with FLUKA *MC* simulation, especially for the lighter protons and helium ions. This finding is made evident through visual inspection of DVH plots in Fig. 3, as well as the dose profiles in Fig. 4. Quantitatively, agreement of FRoG and FLUKA is demonstrated in the DVH statistics in Supplementary Table 2. The mean absolute deviations in D_PTV_ parameters between FLUKA and FRoG for the four ions (protons, helium, carbon, and oxygen ions, respectively) are as follows: 1.03(±0.26)%, 0.46%, 1.11(±0.47)% and 0.63% for D_95%_, 0.55(±0.26)%, 0.75%, 1.22(±0.25)% and 0.99% for D_50%_, and 0.60(±0.26)%, 0.28%, 0.85 (±0.60)%, and 0.69. Overall, absolute deviations in D_95%,_ D_50%_ and D_5%_ for the four ions were ≲1%. Patient A (skull base chordoma) for protons and patient G (chondrosarcoma) for carbon ions are considerably challenging for an analytical calculation, increasing the aggregate percent dose difference for protons and carbon ions. This result was anticipated considering the extent of heterogeneity of the skull for the proton head case and the large penetration depth (higher beam energy) for the carbon ion prostate case. The latter result agrees with findings from the SOBP tests in the homogenous phantom regarding the limitations of Gaussian parameterization. Although FLUKA simulation of the HIT beamline has been validated against physical measurements and found to be in agreement within 1–2% for the four ions^25,26^, foci evolution in air in FLUKA has not been extensively studied. A better comparison of FRoG’s calculation engine and FLUKA would involve incorporating *MC* beam evolution data into FRoG for interpolation of foci values at the source-to-skin distance (SSD) instead of the database of experimental measurements. Despite this issue, agreement was excellent considering FRoG’s calculation times were <4 minutes, as opposed to several hours required for *MC* codes^14^.

The two main reasons for discrepancy between an analytical calculation and FLUKA *MC* simulation are as follows: limitations due to a simplified lateral dose evolution model, as shown in Fig. 2 in the SOBP cases, and inadequate description of PB deformation in heterogeneous conditions when employing techniques such as PB splitting^12^. The effect of the former in the case of protons can be considered less pronounced than for the heavier ions, attributed to increased dose distribution complexity stemming from nuclear interactions. As for the latter, PB splitting attempts to improve lateral range variation agreement; however, improvements in accuracy are restricted by the model’s fixed lateral spread parameters such as Gaussian sigmas and weights of the decomposed beamlets, spreading dose tangentially from the central axis as the homogenous PB model in water.

Unlike the dose calculation assessment in a homogenous phantom which served to scrutinize ray tracing procedures and lateral dose parameterization methods, dose calculation with patient datasets assessed the performance of the PB model and splitting method in heterogeneous anatomy. In most cases, FLUKA and FRoG line profiles (Fig. 4) and DVH plots (Fig. 3) are in excellent agreement. For proton patient cases, slight discrepancies between FLUKA and FRoG occurring at the distal edge can be attributed to the accuracy limits of PB splitting in describing lateral PB distortion. For the heavier ions, D_PTV_ deviations occur in cases with higher penetration depth, where Gaussian parametrization showed $|\triangle |$ ≤ 2% in the SOBP cases. These findings are evident in dose maps and DVHs in Fig. 3, and DVH statistics in Supplementary Table 2. As for OAR volumes, dose deviations between FLUKA and FRoG could be influenced by *MC* statistics. By performing a comprehensive validation for all four ions with various treatment types, FRoG can be cleared as an efficient means of dose calculation in future large-scale retrospective studies to investigate tumor control probability and normal tissue complication probability related indicators in light and heavy ion therapy. With a constant RBE of 1.1 accepted as the clinical standard worldwide for protons, validation of the physical dose engine is adequate; however, for carbon ions, validation of FRoG coupled with biological dose models is a necessary next step in development.

FRoG’s fast yet computationally intensive patient recalculations were made feasible by a GPU-based architecture. Over the last decade, a surge in compact, affordable computational systems like the GPU have found their way into scientific research to perform parallelized computation as opposed to the traditional CPU cluster^20^. As Moore’s law persists, demand for IT and computer science specialists in radiation oncology clinics and medical physics related fields will endure. FRoG’s analytical dose calculation accuracy on the other hand stems from the dual pencil-beam (DPB) model in conjunction with superposition PB splitting. Past approaches to PB splitting are based on a single Gaussian model. This work marks the first technique to separately handle multiple Gaussians during PB splitting, made possible by FRoG’s inherently *MC*-driven physics database. The DPB model is suitable for treatment centers seeking a PB model to best describe the complexity of the pristine and scattered beams separately, such as beam interference from high atomic number (Z) materials in the BAMS which can produce significant large angle particle scattering. Other monitoring systems from proton and heavy ion centers in Europe like CNAO and PSI involve strip ionization chambers for on-line beam position verification^33,34^. The necessity of a novel pencil beam model for such beamlines has yet to be investigated, although BAMS comprised of lower Z materials may have a lessened impact on beam pristineness.

**Figure 5:**
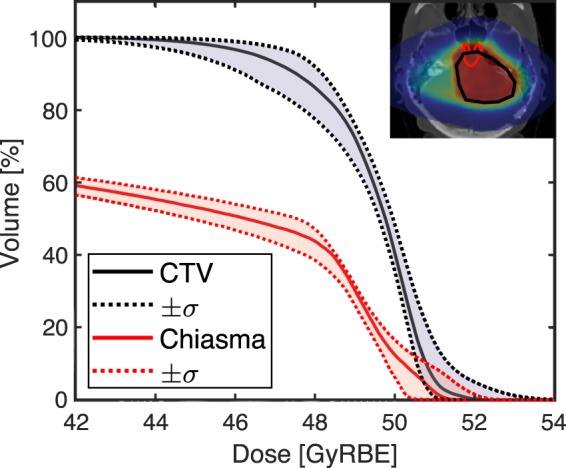
FRoG robustness dose calculation of a proton patient (B), accounting for upper and lower limits of beam spot tune size for the fixed beam rooms at the isocenter (σ = ±10%) for the CTV and an OAR (chiasma).

As previously mentioned, FRoG calculation times were less than 4 minutes for the four ions, fast enough to support clinical activity. Further improvements will be made to FRoG’s calculation speed by upgrading hardware and optimizing code structure. With such improvements, more computationally intensive dose calculation protocols could become commonplace in the clinic, for example, the determination of the planned versus delivered dose to the patient, which would involve dose computation on a fractional basis. Typically, patients receive ~30 fractions within a proton treatment course. Recent works assess the quality and stability of proton treatment delivery using patient-specific machine log files^35^. Similar efforts are made possible with FRoG by utilizing beam parameter measurements (recorded in log files each fraction throughout a patient’s treatment course), shedding light on the confines of patient specific quality assurance (QA) and treatment delivery. The DVH in Fig. 5 depicts forward calculations of patient A using the original physical plan as well as the inter-fractional upper and lower bounds of the beam spot tune size (focus), one of several physical parameters recorded in post-treatment delivery log files. Examination of dose deviation and comparison of DVH bounds demonstrate the value of robust planning, taking into account physical uncertainties or treatment room specific (i.e. fixed beam or gantry) fluctuations into dose calculation. Shifts in D_50%_ and D_5%_ are roughly ±0.3% and ±1.4% for the clinical target volume (CTV), while for the chiasma, calculated shifts were ±2.9% and ±1.5%.

In addition, implementation of biophysical RBE models, including the local effect model (LEM)^36^ and the microdosimetric kinetic model (MKM)^37^ for carbon ions, as well as research driven models^38–40^ for protons and helium ions is ongoing. In regards to carbon ion treatments, FRoG’s multi-model functionality makes the comparison of the two clinical biophysical models used worldwide feasible: MKM at the Japanese facilities (such as the National Institute of Radiological Sciences) and LEM at the European centers, affording a unique inter-comparison of the clinical outcomes applying the two biological perspectives^41^. Lastly, FRoG’s applicability extends to the anticipated helium ion-beam therapy program at HIT. Previously, helium ion beams were pioneered at Lawrence Berkley Laboratory and used to treat over 2000 patients^42^. Without a commercial TPS available for helium ions, in-house developed softwares like FRoG can support the investigation of untapped clinical aspects of radiobiology towards the first patient treatment.

## Conclusion

In this work, the two-part validation of FRoG, a forward calculation engine for proton, helium, carbon and oxygen ion beams, was performed via dosimetric comparison with FLUKA *MC* prediction. First, 3 cm × 3 cm × 3 cm SOBP plans tested the forward recalculation in homogenous settings. Lastly, select patient cases were calculated in FRoG and validated against FLUKA *MC* simulation, evaluating FRoG’s DPB in conjunction with multi-Gaussian PB splitting. Both studies demonstrated FRoG’s excellent agreement with FLUKA *MC* within a timeframe suitable for the clinic, comparable to existing GPU-based systems. In preparation for clinical translation and large-scale patient studies, future endeavors include implementation of research and clinical RBE models as well as the validation of other physical elements such as dose-averaged linear energy transfer.

## Methods

### Platform Architecture

For FRoG, the pencil-beam algorithm^43^ with GPU optimized Siddon raytracing^44^, which provided runtime reductions by up to a factor of 6 in previous works, was employed into the framework. To extract medical data inputs necessary for the dose algorithm, the Python programming language along with packages and toolkits for DICOM and GPU compatibility were implemented. Platform features include physical and biological dose calculation using DG^45,46^ or TG^29,30^ parameterization with the base data generated via FLUKA *MC* simulation^47–49^. All GPU-based recalculations in FRoG were performed on a high-end consumer grade graphics card (NVIDIA Tesla V100). Raytracing is performed at the resolution of the planning CT, whereas dose can be calculated on a downsized grid, following the clinical procedure at HIT. For head treatments, dose calculation is performed on a 2 mm × 2 mm × 2 mm grid with a 0.61 mm × 0.61 mm × 3 mm CT resolution, while for pelvic treatments, dose calculation is performed on a 3 mm × 3 mm × 3 mm grid with a 0.97 mm × 0.97 mm × 3 mm CT resolution.

To achieve short calculation times, the dose algorithm was designed to maximize register and L1-memory (shared memory) usage. Even though an alternating slice approach is used to avoid memory conflicts from racing conditions in L2-memory (global memory) and maximal parallelization is ensured by input pre-processing, the actual dose computation has the highest relative computation time of up to 80% of the overall GPU calculation time, while raytracing and pre-processing take as little as 1% and ~10%, respectively. On average, the FRoG engine spends 10% and 90% of the calculation time performing CPU- and GPU-based processes, respectively, with the GPU to CPU time ratio scaling with the number of pencil beams, as well as other factors (calculation grid size, particle type, etc.). When using higher order lateral parameterization models for improved accuracy like TG in place of DG, the computation time increases by a factor of ~3.

### Particle beam database

The FRoG physics database was generated *in silico* with the FLUKA *MC* development version 2016 for all four ions available at HIT, which incorporated a detailed geometry of the HIT beamline^50^. The HADROTHErapy default setting was selected, with a particle transport and delta ray production threshold set to 100 keV. The production and evaporation of energetic heavy fragments was activated via the COALESCE and EVAPORATION cards, respectively. Patient dose calculations with helium, carbon and oxygen ions required application of a ripple filter as described in previous works^51,52^.

For physical dose calculation, the FRoG database included parameters such as integral depth dose (IDD), and Gaussian parameters (sigmas and relative weights of the Gaussians, respectively) for both DG and TG parameterization. Parameterization of lateral dose fall-off as a function of depth was performed with the MINUIT minimization package^53^ in ROOT^54^ in accordance with least squares fitting^25^.

### Pencil beam model

At HIT, beam characteristics are monitored by the BAMS, which includes a multiple wire proportional chamber (MWPC) composed of a mesh grid of tungsten wiring. The MWPC provides loop-back measurements of the beams position and full width at half maximum (FWHM) in air projected to the isocenter^26^. For the lower Z primary ions (^1^H and ^4^He), interactions in higher Z materials like tungsten can yield large angle scattering as described in previous works^55^. Traditionally, the impact of the MWPC on the particle beam is not explicitly addressed in an analytical TPS whereas FRoG can exploit a mixed-field PB model comprised of pristine beam, scattered particles and fragments. During database generation in FLUKA, primary particles crossing boundaries of the tungsten wiring in the MWPC were flagged. Scoring of these flagged primaries was performed separately from primaries which did not interact with tungsten wiring, yielding a pair of pencil beams for each energy in the HIT database. In summary, DPB model was constructed for proton and helium ions, while a conventional single PB model was implemented for carbon and oxygen ions.

To account for variable lateral dose evolution in the presence of anatomical heterogeneity, PB splitting was incorporated into FRoG’s framework following the mathematical procedures of beamlet superposition^56^. In a 2D grid space, the splitting method involves a superposition of N equally-spaced sub-Gaussians (bounded by ±3.5σ) with equivalent FWHM but variable weighting. In this work, beamlet superposition was performed for all calculations to maximize the splitting multiplicity in heterogeneous conditions. For protons and helium ions, ~700 sub-splits were used when implementing the DPB model, while for carbon and oxygen ions, ~350 subs-splits were performed using a single PB model. For each beam spot in the patient plan, SSD was calculated and FWHM values in air projected to the entrance were interpolated using experimentally measured FWHM values for each ion, beam energy, foci and treatment room (fixed-beam or gantry) as in clinical practical at HIT using Syngo.

**Figure 6:**
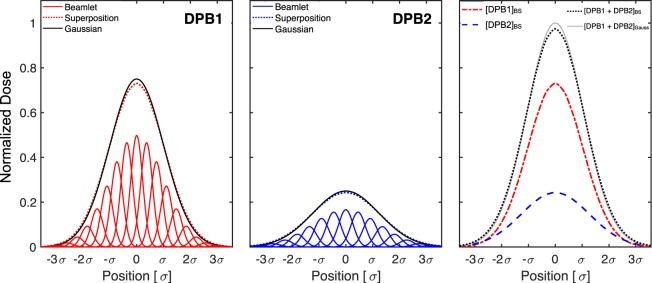
Dual PB (DPB) is implemented in FRoG for ^1^H and ^4^He by separately scoring for particles interacting with the MWPC versus the pristine beam. Beamlets, superposition and fully reconstructed Gaussian for the primary PB (left) and the secondary (scattered) PB (middle), with final superposition (aggregate) of primary and secondary PBs (right). A 1D splitting multiplicity of M = 21 (analogous to the 2D case in FRoG) is depicted, yielding <1% reduction of maximum dose of the aggregate PB from original Gaussian (with integral dose conserved).

For proton and helium cases, the DPB model accounting for interactions in the BAMS was implemented and is depicted in Fig. 6. IDD ratio of particles interacting in the BAMS to the pristine primary particles was ~0.25. The second weighting factor for the secondary PB measured in air at the isocenter varies from roughly 22% to 24% and 17% to 24% for all available proton and helium ion beam energies, respectively. As a first approximation, the effect of the MWPC on the PB is neglected for the heavier ions, carbon and oxygen, since the degree of large angle scattering decreases with particle mass, resulting in reduced second and third Gaussian sigmas and weights. Therefore, a conventional PB model using a single database was justified for carbon and oxygen ions.

### Platform validation in a homogenous water phantom

For preliminary testing and characterization, FRoG forward dose calculations were performed in QA-mode (without PB splitting) for a homogeneous water phantom with the surface situated at isocenter. SOBP plans for the four ions were generated and optimized using the MCTP following the same procedure as described in the recent validation^25^. During optimization, rectangular parallelepiped structures (3 cm × 3 cm × 3 cm) were delineated and centered at three depths: 50 mm, 125 mm and 200 mm. FRoG’s forward calculations of SOBP plans were comprehensively evaluated against the FLUKA *MC* simulated data, previously validated against dosimetric measurements^26,28,46,57^. The following dosimetric parameters to characterize depth and lateral dose evolution were extracted: R_80_, TPR, EPR, DFO_80/20_, LFO_80/20_, H_D_, $\bar{D}$, and $| \% \triangle |$. Even for a commercial TPS, the target size chosen (3 cm × 3 cm × 3 cm) represents a challenging clinical scenario in a homogenous setting, susceptible to uncertainties and sensitive to physical beam specifications (e.g. energy, focus and step size)^58,59^.

### Performance in heterogeneous cases (patients)

For evaluation of FRoG’s forward calculation with patient cases, *MC* was executed for comparison using an in-house FLUKA simulation protocol known as FICTION^14^. The in-house CPU cluster containing ~300 nodes (Intel Xeon CPU E5-2683 16 core @ 2.1 GHz) was used for each patient calculation, running between 1% and 6% of the total primary particles in the plan, with the clinically applied dose grid (described in Table 1)^14^. All proton and carbon ion patients presented have been biologically optimized with the clinical TPS at HIT, Syngo RT Planning (Siemens, Erlangen, Germany), which allowed direct simulation using the FICTION framework, whereas helium and oxygen research beams cannot be biologically optimized with Syngo. Additional proton and carbon ion patients were selected for helium^60^ and oxygen ion treatment planning, respectively, and biological optimized plans were generated via MCTP^27,61^. Optimization was performed with a recently developed phenomenological model for helium RBE prediction^38,40^ and an adapted LEM-I model for Oxygen, after model interfacing with FLUKA^52^. Information regarding the selected patient cases is displayed in Table 1. Physical dose calculation for each patient was subsequently executed in FRoG following the PB splitting procedure for TG parameterization. After forward calculation using FRoG and FLUKA, DVHs and dose line profiles were generated for each case. Various clinical endpoints were investigated, including D_95%_, D_50%_, and D_5%_.

Patients records were obtained with informed consent and handled following the Helsinki Declaration. All methods were approved and followed applicable guidelines and regulations of the Heidelberg University Medical Faculty. Considering the retrospective nature of the study, clearing from the ethical review committee was not required. All records were anonymized prior to the study.

## Electronic supplementary material

Dataset 1

Dataset 2

Dataset 3
